# Bioinformatics and Computational Biology in Poland

**DOI:** 10.1371/journal.pcbi.1003048

**Published:** 2013-05-02

**Authors:** Janusz M. Bujnicki, Jerzy Tiuryn

**Affiliations:** 1 Laboratory of Bioinformatics and Protein Engineering, International Institute of Molecular and Cell Biology in Warsaw, Warsaw, Poland; 2 Bioinformatics Laboratory, Institute of Molecular Biology and Biotechnology, Faculty of Biology, Adam Mickiewicz University, Poznań, Poland; 3 Faculty of Mathematics, Informatics and Mechanics, University of Warsaw, Warsaw, Poland; National Center for Biotechnology Information (NCBI), United States of America

The series of articles in *PLOS Computational Biology* on the development of bioinformatics activities in various countries, e.g., China [1], Australia [2], and Singapore [3], and the formation and successful development of the Polish Bioinformatics Society over the last five years, have inspired us to present a personal perspective on the advances of bioinformatics in Poland.

## Origins and History

The development of bioinformatics in Poland has built primarily on two scientific disciplines: mathematics and computational sciences, and biological physics including protein crystallography.

Mathematics has been strongly established as a research discipline for several decades in Poland, providing a basis for the more recent development of excellence in computer science. The Polish School of Mathematics, as it is called today, refers to the community of Polish mathematicians during the period between the two twentieth century World Wars. It consisted of three schools related to the cities of their location: Cracow (Stanisław Zaremba), Lwów (Stefan Banach) (this city at present belongs to Ukraine and it is named Lviv), and Warsaw (Wacław Sierpiński). During World War II, Poland lost about 50% of its mathematicians by death or emigration [4]. However, a traditionally very strong Lwów-Warsaw School of Logic (Alfred Tarski, Jan Łukasiewicz, Andrzej Mostowski), with the Lwów University moved to Wrocław (former German Breslau), has spawned, starting in the 1970s, Polish research groups strong not only in theoretical computer science but also in computer software development. It is worth mentioning that Hugo Steinhaus, a Polish mathematician, who apparently “discovered” Stefan Banach in 1916 in Cracow, had a prominent role in building the Polish School of Mathematics after World War I, and then helped to restore the Polish mathematics community after it was severely crippled during World War II.

A prominent role in applying mathematical methods to biological questions was played by Polish mathematician Stanisław Ulam. He was born in Lwów in 1909, and later emigrated to the United States. From 1968 until his death in 1984, Ulam held a position of professor of biomathematics at the University of Colorado School of Medicine. He was interested in a very broad spectrum of pure and applied areas of mathematics. It is interesting to note that, among many other achievements, he was involved in the Manhattan Project at Los Alamos and that he was the founder of the very popular Monte Carlo method, widely used in bioinformatics. Since 1997, in recognition of his contributions to the area of computational biology, the annual conference RECOMB (Research in Computational Molecular Biology) has established a prestigious series of Ulam Lectures, which are intended to be less technical and more casual.

The first department of biophysics in Poland was organized by David Shugar in 1966 at the Faculty of Physics, University of Warsaw (UW), with the support of Leopold Infeld from the Institute of Theoretical Physics UW and Jerzy Pniewski from the Institute of Experimental Physics UW. Another center of biophysics research was established in the Institute of Biochemistry and Biophysics (IBB), Polish Academy of Sciences (PAS), created already in the 1950s in Warsaw by Józef Heller. The first molecular biophysics studies in these institutions were focused on nucleic acids and their building blocks. Studies conducted in the 1960s and early 1970s by Polish researchers or with their participation, which laid ground for the future computational analyses, ranged from comparative analyses of homologous sequences [5] to studies of conformations of nucleosides and nucleic acids [6]. Interests of some of the researchers involved in these analyses evolved toward theoretical analyses of nucleic acid bases, e.g., [7], and were further were extended to larger biomolecular systems such as nucleic acids, proteins, and their interactions [8], [9].

Protein crystallography in Poland was initiated shortly before World War II by Tadeusz Baranowski, who grew protein single crystals of muscle myogen (review: [10]). During the politically difficult decades between the end of World War II and the fall of the communism in Central Europe, in particular in the 1970s and 1980s, a host of Polish crystallographers left the country or decided to stay abroad for various reasons. Today many of them (too many to mention in this short article) are found at senior positions in various institutions, including protein crystallography beamlines at synchrotron facilities, in particular in the United States and in the United Kingdom. Among macromolecular crystallography laboratories that exist in Poland today, the first one, the Center for Biocrystallographic Research, led by Mariusz Jaskólski, was created only in 1994 in the Institute of Bioorganic Chemistry (IBCh) PAS. At the beginning of the twenty-first century, more macromolecular crystallography laboratories were created in Poland, with particular concentration in Warsaw and Poznań. Polish macromolecular crystallographers, both in Poland and abroad, form a closely knit community, sometimes referred to jokingly as the “Polish crystallographic mafia.”

It is worth mentioning the influence of Wacław Szybalski, who can be considered one of the founding fathers of synthetic biology. In 1949, after earning a PhD in chemistry from the Institute of Technology in Gdansk, he emigrated first to Denmark and then to the United States, where he has worked on genetics and bioengineering at the Cold Spring Harbor Laboratory, Rutgers University, and then the University of Wisconsin-Madison. He was the first to insert DNA into human cells and developed various tools and methods in molecular biology. Importantly, he has always been a strong and concrete supporter of science in Poland.

While protein crystallography and computer science provided strong scientific basis for the development of structural bioinformatics in Poland, the first generation of Polish bioinformaticians comprised two lineages: one with origins mostly in the field of macromolecular biophysics and simulations of polymer folding and the other in computational analysis of biological sequences as strings.

Polish researchers have been traditionally very active in protein structure prediction by computational techniques. In particular, Andrzej Koliński has extended his studies on polymer folding by Monte Carlo dynamics simulations done in the 1970s and early 1980s [11] to analyzing protein folding in solution. In collaboration with Jeff Skolnick in the United States (at Washington University in St. Louis, later at the Scripps Institute, San Diego) he extended this approach, with the use of coarse-grained models and statistical potentials [12], to what currently constitutes one of the most successful computational approaches to protein folding. Adam Godzik, together with Koliński and Skolnick, has coinvented another approach for protein structure prediction, based on threading the protein sequence along the backbones of known structures, so these structures could be used as templates in comparative modeling [13]. The Monte Carlo methodology has been independently used to simulate protein folding by Adam Liwo at the University of Gdańsk in collaboration with Harold Scheraga at Cornell University in Ithaca (United States) since the early 1990s [14]. These general approaches have provided the foundation for the development of some of the most successful contemporary methods for protein structure modeling, and they are also being used for modeling of RNA and various macromolecular complexes by many groups in different countries around the world.

As a result of political and economic changes in Poland after the fall of the communist system in 1989, the science funding scheme that used to be fully based on central planning has underwent a significant change, including the establishment of new research funding agencies (both governmental and nongovernmental) that started awarding grants based on open competitions. These changes in the system coincided with the boom in genomic sequencing as well as the development of the internet, facilitating the free availability of data generated all over the world. As a consequence, bioinformatics has started expanding more rapidly in Poland. For instance, in 1993 Marek Niezgódka and Bogdan Lesyng created the Interdisciplinary Centre for Mathematical and Computational Modelling (ICM) at UW. The infrastructure included the first CRAY computer in Middle-Eastern Europe. The main areas of research and education included multiscale biomolecular modeling, such as the combination of quantum and classical dynamics simulations of enzymatic reactions. Some of the earliest work in this area was carried out by researchers from ICM in collaboration with J. Andrew McCammon at the University of California, San Diego [15]. In 1994, the Polish node of the European Molecular Biology network (EMBnet) was established by Piotr Zielenkiewicz in IBB PAS and offered access to major macromolecular sequence and structure databases together with a variety of analysis tools as a basis of bioinformatics training and education.

In the middle of the 1990s, Polish researchers in the United States (in particular Krzysztof Fidelis and his coworkers, then at Lawrence Livermore National Laboratory) were involved in the development of the Critical Assessment of techniques for protein Structure Prediction (CASP) initiative, headed by John Moult at the Center for Advanced Research in Biotechnology (CARB) in Rockville, Maryland [16]. The introduction of this large-scale experiment to assess methods for protein structure prediction in the course of a blind analysis (where the experimentally determined structures are not known to modelers at the time of modeling) has transformed the field of bioinformatics and sparked other analogous initiatives in the whole field of computational biology [17]. CASP has also become a forum for Polish bioinformaticians to present their successful developments.

At the beginning of the twenty-first century, Leszek Rychlewski (a former postdoc of Adam Godzik) and his coworkers (including one of the authors of this article, Janusz Bujnicki, then Rychlewski's graduate student) significantly contributed to the development and popularization of meta-prediction in the area of template-based structure prediction [18], [19]. Ultimately, meta-prediction and coarse-grained modeling were extremely successful in CASP-5 and CASP-6 in 2002 and 2004, where three Polish researchers and their groups (Krzysztof Ginalski, Andrzej Koliński, and Janusz Bujnicki) were placed among the five top-scored groups in protein 3D structure prediction, and where the application of the 3D-Jury meta-prediction (invented by Rychlewski) and similar approaches were shown to significantly improve individual primary predictions made by human experts and automated servers alike [20]–[23].

The second lineage of Polish bioinformaticians emerged at the end of the 1990s in the field of computational analysis of DNA and protein sequences. Jacek Błażewicz at the Poznań University of Technology (PUT) in Poznań (later also at the IBCh PAS) and one of the authors of this article (Jerzy Tiuryn) were computational scientists who became interested in computational biology and established research groups in the area of bioinformatics. Scientific cooperation between these two groups was implemented through regular annual meetings, where PhD students presented progress in their research. These meetings from 2009 onward gave rise to annual PhD workshop meetings conducted on the national level under the umbrella of the Polish Bioinformatics Society. Initially, the research interest of both groups was focused on sequence analysis. To give a flavor of the problems investigated, we mention some papers published in this subarea: the Błażewicz group started with research on sequencing by hybridization [24], while the Tiuryn group introduced and studied the so-called contextual alignments [25]. The reader may notice an alphabetical order of authors in both articles, which was then typical for the computer science community.

Independently, in 1995, Stanisław Cebrat together with Mirosław Dudek, a physicist from the University of Wrocław, founded a group that focused on the development and application of algorithms for analyses of protein-coding sequences (e.g., [26]), global structure and organization of genomes, and simulations of population evolution, including age and genetic structure.

## Main Bioinformatics Research Centers in Poland

**Figure 1:**
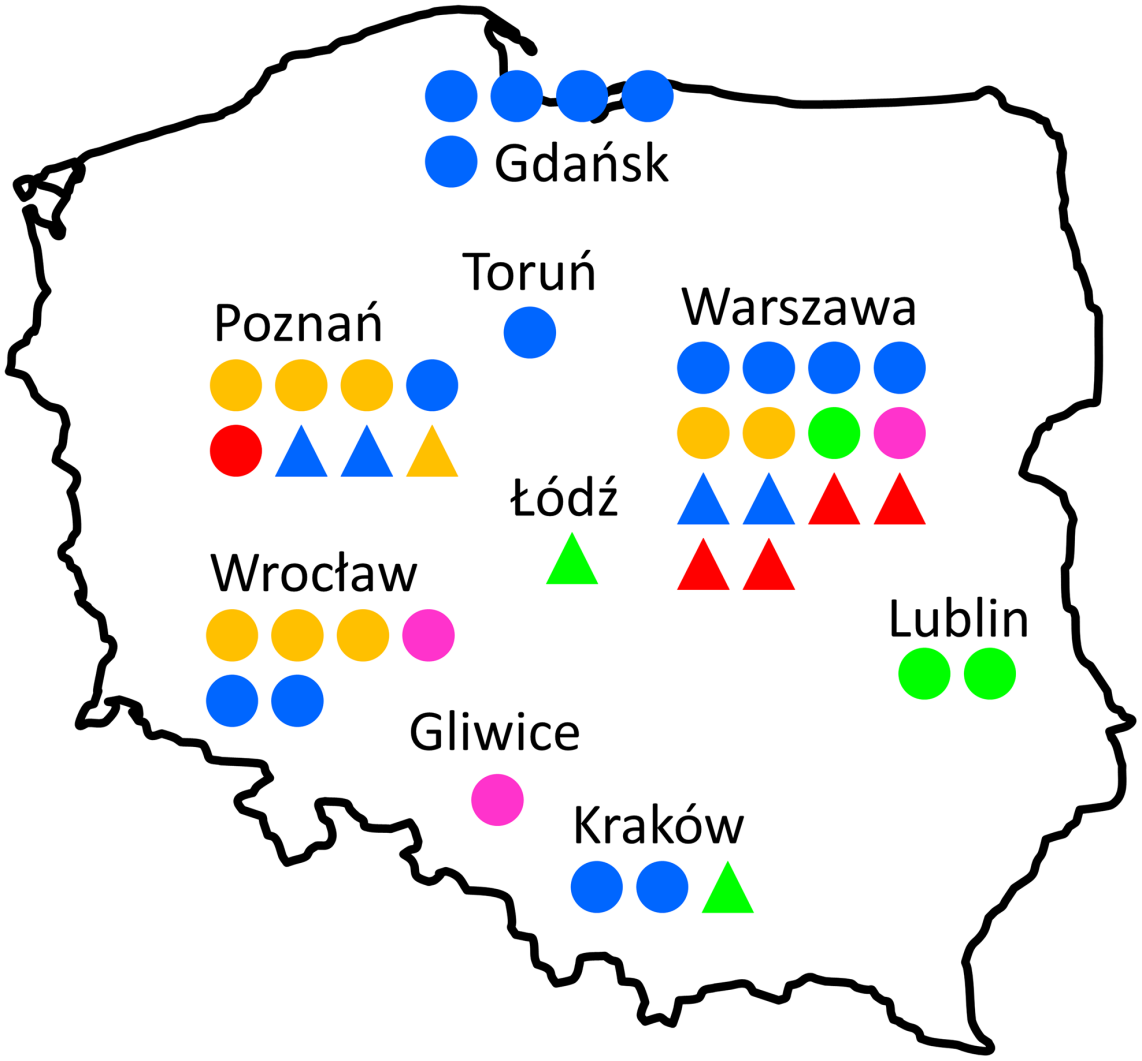
Geographical distribution of computational biology research groups in Poland mentioned in this article. Groups located in universities (also medical or technical universities) are indicated by circles, and groups located in other institutions (institutes of Polish Academy of Sciences, International Institute of Molecular and Cell Biology in Warsaw, and BioInfoBank in Poznań) are indicated by triangles. Colors indicate the dominant research topic of each group: blue, focus on macromolecular structure and dynamics (research on proteins, nucleic acids and large molecular assemblies, structure prediction, molecular simulations, etc.); green, focus on small molecules (including protein-ligand interactions, virtual screening); orange, focus on –omics (genomics, transcriptomics, proteomics); red, focus on systems biology; magenta, focus on development of algorithms, machine learning and/or data mining.

Today the field of bioinformatics in Poland is characterized by a variety of topics and research methods used. Undoubtedly, the most popular topics are still protein structure prediction and analysis, followed by biological sequence analysis. Emerging new topics include analyses of biological data produced by new high-throughput experimental methods, systems biology, and modeling of RNA structure and interactions. Here, we will briefly list major research groups led by independent researchers that have been active in the field of bioinformatics for at least several years, organized according to their current geographical locations. Figure 1 illustrates the distribution of these groups according to academic centers, type of institutions, and main research topics. This landscape is obviously very dynamic and we expect new groups to appear even in the time frame between the submission and publication of this article, and the research topics are likewise expected to evolve.

### Warsaw (Warszawa)

There is a strong base of bioinformaticians at the University of Warsaw (UW), and we have already introduced several researchers whose groups are active in protein structure prediction. At the Faculty of Chemistry UW, Andrzej Koliński's group is involved in the development of methods for modeling of protein structures and complexes, mostly using a coarse-grained representation and restraints derived from experimental data (see e.g., [27]). Sławomir Filipek's group is involved in the modeling of structure, dynamics and interactions of membrane proteins and their interactions with drugs [28]; e.g., this group in collaboration with the Krzysztof Palczewski group in the United States, then at the University of Washington in Seattle, created models of rhodopsin complexes [29] employed later by theoretical and experimental groups to study dimerization and activation of G-protein–coupled receptors. Krzysztof Ginalski's group at the Centre of New Technologies (CeNT) UW is involved in various aspects of bioinformatics, including remote homology detection and classification of protein superfamilies, in particular enzymes acting on nucleic acids [30], [31]. Dariusz Plewczyński has been pursuing the development of meta-predictors for small molecule docking and virtual screening [32] and for prediction of post-translational modifications [33]. Joanna Trylska's group at CeNT is involved in the development and application of methods for biomolecular simulations of large macromolecular complexes involving proteins, nucleic acids, and small molecules [34]. Witold Rudnicki's group at the ICM UW works on applications of GPU-accelerated algorithms in sequence alignment, as well as on the application of machine learning in various aspects of bioinformatics, such as analysis of drug resistance mutations in HIV-1 reverse transcriptase, in collaboration with Jan Komorowski at the Uppsala University in Sweden [35]. The Faculty of Mathematics, Informatics and Mechanics (MIMUW) hosts the group of Jerzy Tiuryn, whose interests extend beyond the aforementioned sequence analyses, and include phylogenetics and evolutionary biology with emphasis on the evolution of gene families [36], inferring phylogeny from whole genomes [37], and comparative genomics [38]. They have also pursued systems biology, with emphasis on investigation of signaling pathways [39], [40] and protein-protein interaction networks [41]. Bartek Wilczyński, Tiuryn's former apprentice, now leads an independent group working in the area of computational genomics, e.g., on computational enhancer prediction [42], [43] and Bayesian network modeling of gene regulatory networks [44]. He has also been involved in the development of Biopython [45]. Systems biology focused on applications in molecular medicine is the main research area of the group led by Anna Gambin (who is coaffiliated with the Mossakowski Medical Research Centre PAS). Their spectrum of interests covers clinical degradomics [46], kinetic modeling of oncogenic pathways [40], and processing of mass spectrometry data [47]; they also cooperate with clinicians from the Baylor College of Medicine (Houston, United States) on analysis of genomic disorders [48].

There are also groups involved in computational biology research at other institutions in Warsaw. Piotr Zielenkiewicz directs IBB PAS and his group is involved in many different activities, such as research on text mining [49] and computational modeling of protein-protein [50] and protein-drug interactions [51]. Bogdan Lesyng at the Mossakowski Medical Research Centre (MMRC) PAS leads a group involved in the development and applications of bioinformatics and systems biology methodologies in molecular medicine, focused on theoretical and experimental studies of oncogenic processes, including multiscale modeling, drug design, microarray analyses, and simulations of oncogenic signaling pathways. As an example, in collaboration with Waldemar Priebe at the MD Anderson Cancer Center (Houston, United States) and Bozena Kamińska at the Nencki Institute of Experimental Biology PAS, they studied small molecule regulators of the JAK/STAT oncogenic regulatory network [52]. Marek Cieplak and the Institute of Physics PAS is involved in theoretical research on protein folding and unfolding (in particular by stretching) using molecular dynamics and lattice models [53], [54]. Lucjan Wyrwicz's group at the Maria Skłodowska-Curie Memorial Cancer Centre in Warsaw is involved in various aspects of bioinformatics, particularly in relation to cancer and oncology [55]. Tomasz Lipniacki's group at the Institute of Fundamental Technological Research (IPPT) PAS is involved in research on computational systems biology, in particular stochastic simulations of regulatory systems [56]. International Institute of Molecular and Cell Biology in Warsaw (IIMCB) hosts a group of one of the authors of this article (Janusz Bujnicki), which is working on a variety of topics, including the development of predictive methods and their application in, e.g., protein structure prediction [57], prediction of intrinsic disorder in proteins [58], and prediction of protein-RNA interactions [59]. They are also involved in comparative bioinformatics of protein superfamilies [60] and in experimental protein engineering guided by bioinformatics [61]. Another area of their research is the development of databases of information about pathways of nucleic acid metabolism [62].

### Poznań

Bioinformatics research is also strong in the city of Poznań, and it is worth emphasizing that it is dominated by work related to RNA. Four computational biology groups are hosted by the Faculty of Biology at the Adam Mickiewicz University (AMU) within the Laboratory of Bioinformatics organized by Artur Jarmołowski and Zofia Szweykowska-Kulińska. Izabela Makałowska's group is involved in the computational analysis of gene and genome evolution [63]. Wojciech Karłowski's group is working on computational genomics, including annotation and functional analyses of small RNAs [64]. Borys Wróbel's group (who is coaffiliated with the Institute of Oceanology PAS in Sopot) is involved in computational simulation of development of multicellular organisms [65]. AMU also hosts a group under supervision of Janusz Bujnicki, focused on the development of tools for RNA modeling [66] and structural bioinformatics analyzes of enzymes acting on RNA [67].

In Poznań, there are also several groups associated with PUT or IBCh PAS, or both, and involved in computational biology research. Jacek Błażewicz directs a large team of researchers, some of whom are affiliated with both institutions. It comprises several groups working on various aspects of computational biology: Marta Kasprzak and Aleksandra Świercz are working on DNA sequencing, assembling and mapping [68] and on microarray data analysis. Piotr Formanowicz conducts research on the application of Petri nets to study metabolic processes [69]. Piotr Łukasiak is working on protein structure modeling and evaluation (i.a., in cooperation with Krzysztof Fidelis, now at the University of California, Davis, United States). Marta Szachniuk designs algorithms for RNA structural bioinformatics. Paweł Wojciechowski is developing GPU-based algorithms for various aspects of sequence alignment and next-generation sequence data analysis [70]. The Błażewicz group is also working on other aspects of computational biology, such as modeling of RNA metabolism [71] and modeling of viral infections [72]. Ryszard W. Adamiak's group at IBCh PAS is working on biomolecular NMR (Zofia Gdaniec) and molecular dynamics simulations (Tadeusz Kuliński) of RNA, and in collaboration with the Błażewicz team (with Marta Szachniuk), they are involved in the development and application of tools for RNA 3D structure modeling [73], [74], and in NMR spectra analysis. Jan Barciszewski's group at IBCh PAS has developed databases of non-coding RNAs [75] and of aminoacyl-tRNA synthetases [76]. Last but not least, Leszek Rychlewski, who was mentioned earlier in this article, has founded BioInfoBank, the first bioinformatics-oriented enterprise (SME) in Poland, which was initially involved in the development and application of tools for protein structure prediction and now focuses on the design of therapeutic proteins and drugs [77] and on technology transfer.

### Wrocław

Computational biology groups are also active at different institutions in Wrocław. At the Faculty of Chemistry of the Wrocław University of Technology (WRUT), a group led by W. Andrzej Sokalski is involved, i.a., in the development of new computational methods: the Differential Transition State Stabilization (DTSS) approach for exploring origins of enzyme catalytic activity, analysis and prediction of activity of enzyme inhibitors, and the Catalytic Field technique for rational design of biocatalysts [78]. Olgierd Unold and his coworkers at the Faculty of Electronics of WRUT are working on the application of machine learning techniques in bioinformatics, e.g., prediction of amyloidogenic regions in proteins [79]. At the Faculty of Fundamental Problems of Technology of WRUT, a group led by Małgorzata Kotulska is working in the field of structural bioinformatics, e.g., the quality assessment of models of protein 3D structure [80]. At the same faculty, a group led by Małgorzata Bogdan works in the area of statistical human genomics, in particular on the problem of locating quantitative trait loci (QTL) [81]. At the Faculty of Biology and Animal Science of the Wrocław University of Environmental and Life Sciences, Joanna Szyda's group works on statistic genomics of animals [82]. Stanisław Cebrat's group at the Faculty of Biotechnology of the University of Wrocław is working in the area of theoretical and computational genomics, e.g., on Monte Carlo simulations of genome and population evolution [26], [83].

### Gdańsk

In Gdańsk, a number of researchers are working on the boundary of computational biology and computational chemistry. At the Faculty of Chemistry of the University of Gdańsk (UG), Adam Liwo and Cezary Czaplewski with their groups continue the aforementioned work on coarse-grained modeling of protein and peptide folding in collaboration with the Scheraga group in the United States [84], [85]. Jerzy Ciarkowski's group works on molecular dynamics of proteins and peptides, in particular those associated with biological membranes [86]. A group led by Sylwia Rodziewicz-Motowidło is working on various aspects of protein and peptide folding, in particular on cystatin C [87]. At the Intercollegiate Faculty of Biotechnology of the UG and the Medical University of Gdańsk (GUMED), there are two groups: one led by Stanisław Ołdziej, who studies, e.g., conformational transitions in proteins by molecular dynamics [88], and one led by Rajmund Kaźmierkiewicz, who studies molecular interactions, e.g., in bacterial kinases responsible for biofilm formation [89].

### Cracow (Kraków)

In Cracow, a group at the Faculty of Biotechnology of the Jagiellonian University (JU) led by Marta Pasenkiewicz-Gierula focuses on the application of molecular dynamics simulations in the study of lipid bilayers and their interactions with various membrane-active molecules [90]. Irena Roterman-Konieczna's group at the Collegium Medicum JU is working on various aspects of structural bioinformatics, e.g., protein folding and interactions [91]. A group led by Andrzej J. Bojarski at the Institute of Pharmacology PAS is mainly known for its studies on the serotonin receptors; their research involves a variety of computational techniques, such as conformational sampling in homology modeling [92], virtual screening, virtual combinatorial library design and analysis, as well as measuring ligand-protein interactions [93].

### Other cities in Poland

There are numerous other research groups with interests close to computational biology in other cities in Poland. One bioinformatics center exists in Gliwice; groups led by Andrzej Polański and Andrzej Świerniak at the Silesian University of Technology in Gliwice collaborate with the Maria Skłodowska-Curie Memorial Cancer Centre and Institute of Oncology (a state-owned medical research institute), and are involved in research on analysis of gene expression [94], [95] and proteomics data [96]. Wiesław Nowak at the Nicolaus Copernicus University (NCU) in Toruń is working in the area of biomolecular simulations of conformational transitions and interactions of enzymes with small molecules [97]. A group headed by Marek Cypryk at the Centre of Molecular and Macromolecular Studies PAS in Łódź is working, i.a., on computational chemistry and modeling of the chemical reaction systems [98]. Groups led by Dariusz Matosiuk and Krzysztof Jóźwiak at the Medical University of Lublin extensively use methods of bioinformatics and cheminformatics for modeling of proteins, in particular membrane receptors, and their interactions with small molecule ligands [99], [100].

## Bioinformatics Education in Poland

There are a growing number of universities that have started undergraduate programs in bioinformatics, computational biology, and related disciplines in response to the growing demand for undergraduate education in these areas. The first BSc/Msc program in bioinformatics (2+3 years of study) was introduced at the Faculty of Biology, AMU in Poznań in 2003 (currently conducted jointly with the Faculty of Computer Science, PUT). Other BSc/MSc programs in bioinformatics were established in other major academic centers in Poland: in 2005 at the Faculty of Chemistry of WRUT (also in English since 2011); in 2008 at the Faculty of Natural and Technical Sciences of the Opole University; in 2008 at the University of Warsaw as a joint initiative of the Faculties of Biology, Mathematics, Informatics and Mechanics (MIM), and Physics; and in 2009 at the Faculty of Computer Science and Material Sciences of the Silesian University in Katowice. In 2012 a BSc/MSc program in bioinformatics was initiated at the JU in Cracow as a joint initiative of the Faculty of Mathematics and Computer Science, Faculty of Biochemistry, Biophysics, and Biotechnology, and Institute of Environmental Sciences as an extension of courses on molecular modeling and bioinformatics taught at the Faculty of Biochemistry, Biophysics, and Biotechnology since the late 1990s. Many universities have also introduced bioinformatics at the MSc level alone (see, e.g., http://pl.wikipedia.org/wiki/Bioinformatyka). To our knowledge, there is only one PhD school dedicated to bioinformatics in Poland, organized jointly by the Polish-Japanese Institute of Information Technology (a private school), Genomed (a private company), and the Maria Skłodowska-Curie Memorial Cancer Centre and Institute of Oncology. However, it should be acknowledged that many PhD theses in bioinformatics and computational biology are regularly defended at many research and higher educational institutions throughout Poland. It is expected that the number of both undergraduate and postgraduate programs in bioinformatics will grow. Notably, recently (July 2012) the Faculty of Mathematics, Informatics and Mechanics, University of Warsaw jointly with the Institute of Mathematics, Polish Academy of Sciences was awarded the status of the Leading National Research Center (KNOW) in Mathematical Sciences, associated with a grant for supporting education, infrastructure, and research. One of the leading research areas of the center is bioinformatics and computational biology, and planned activities include the expansion of a PhD program that involves bioinformatics.

## Polish Bioinformatics Society—From Training to Cooperation

In the absence of government initiatives and dedicated institutional support, the bioinformatics community in Poland has been quite fragmented and uncoordinated. The field has consolidated in a bottom-up manner, starting with several independent initiatives initially aimed at training students.

The scientific contacts between the Tiuryn and Błażewicz groups have prompted the development of a joint summer school for students, which started in 2003 and has continued on an annual basis ever since. The formula of these meetings consisted of oral presentations by PhD students on their ongoing work. This workshop has later grown to become a regular bioinformatics conference, comprising invited lectures from senior scientists, contributed talks selected from abstracts submitted by junior scientists (preferably PhD students), as well as a poster session. Bioinformatics in Toruń (BIT) is an independent initiative started in 2001 by Wiesław Nowak at the NCU in Toruń, which has evolved from a local workshop dominated by hands-on tutorials for students to an annual international conference with >100 participants. In Poznań, a group of scientists from the Faculties of Biology and Physics of AMU, enthused by Artur Jarmołowski, together with a couple of Polish bioinformaticians then living in the United States (Wojciech Makałowski and Izabela Makałowska) initiated Poznań Summer School in Bioinformatics, which has been organized almost every summer since 2002.

Polish Bioinformatics Society (PBS, or PTBI in Polish) was formed in February 2008 after a period of intense discussions between some of the aforementioned scientists, members of their groups, and other researchers who entered the field of bioinformatics more recently from various disciplines. The first board of PBS, comprising the coauthors of this article (Jerzy Tiuryn as president and Janusz Bujnicki as a vice president), Wiesław Nowak (as a second vice president), Marta Pasenkiewicz-Gierula (as secretary), and Witold Rudnicki (as treasurer) has successfully organized the works of the society, in particular coordinating the organization of events started by local communities. PBS has also organized an annual nationwide competition for the best MS thesis in bioinformatics (since 2009), and then later (since 2010) an annual competition for the best PhD thesis in bioinformatics. Currently, it has >150 members and keeps growing. PBS is currently working toward strengthening interactions with neighboring fields of research, in particular evolutionary biology, biochemistry, and structural biology. In the fall of 2011, Janusz Bujnicki together with a group of Polish crystallographers (Mariusz Jaskólski from Poland and Zbigniew Dauter, Wladek Minor, and Alexander Wlodawer from the United States) have organized a major international conference, “Multi-Pole approach to structural biology,” that has gathered together Polish structural bioinformaticians and macromolecular crystallographers as well as representatives of other disciplines to discuss the advances of structural biology that have been promoted with the participation of scientists with Polish roots and/or with significant links to Poland. The conference has brought the two fields closer to each other, strengthened the existing links, and prompted the development of new collaborations. PBS has also engaged in the organization of a joint congress (planned for 2014) with other Polish learned societies in the fields of biochemistry, cell biology, and biophysics. It is envisaged that more such conferences would be organized in the future, perhaps at the intersections of bioinformatics with other disciplines that are strongly established in Poland, such as computer science or chemistry.

## Toward the Future

Polish computational biologists are recognized in the world and are involved in an extensive network of collaborations with other theoretical and experimental laboratories both nationally and worldwide. The existing host of researchers together make a critical mass that can drive the development of bioinformatics in Poland through a combination of collaboration and amicable competition (e.g., as evidenced by the activities of PBS). Following Poland's accession to the European Union, structural funds (about EUR 4.1 billion for the years 2007–2013) have significantly contributed to the development of scientific infrastructure as well as to the modernization of the higher education sector. Examples of investments into bioinformatics infrastructure include the development of a joint grid of high-performance computing clusters by six institutes (IIMCB, IBB PAS, IPPT PAS, Institute of Experimental Biology PAS, Institute of Clinical and Experimental Medicine PAS, and Institute of Biocybernetics and Biomedical Engineering PAS) on the Ochota Campus in Warsaw, and the European Center of Bioinformatics and Genomics in Poznań (jointly by PUT and IBCh). During the course of the recent reforms of the science funding system in Poland, two state agencies have been created, the National Science Centre (NCN) and the National Centre for Research and Development (NCBiR), that offer competitive grant schemes that can be used (and have been used with success, particularly in the case of NCN) for bioinformatics-related projects. Grants to fund computational biology–oriented projects have been also funded by the Foundation for Polish Science (FNP), an independent, self-financing, nonprofit, nongovernmental organization established in 1991. In 2011, the FNP Award, which is considered the most prestigious national award for scientific research in Poland, went to Andrzej Koliński for the recognition of his achievements in the development and practical application of unique methods for protein structure prediction. All three aforementioned research-funding agencies have successfully introduced special grant programs for scientists early in their careers, aimed at counteracting the brain drain and attracting young researchers to start their independent laboratories in Poland. The development of new laboratories in computational biology is particularly important given the growing needs of the educational sector and the emerging new programs in bioinformatics that require teachers and trainers who are active bioinformatics researchers.

There are, however, significant challenges that need to be overcome. It must be admitted that Poland belongs to the group of the EU member states with the lowest level of investment in research and technology development overall. One of the reasons for this is that access to financial sources other than the state budget is quite limited. In particular, the investments of business entities in R&D activities are rather low, and the Polish fiscal and administrative system could be more supportive of commercializing the results of research with an applied edge. The resources of FNP are relatively scarce, and they by necessity are focused on supporting only the best scientists and research teams. It must be acknowledged that many bioinformatics laboratories and individuals in Poland benefit from grants funded by international sources (in particular from the Framework Programme of the EU). However, further reforms within the national R&D structure and financing system in Poland are required to lift some of the barriers that slow down the development of science in general and interdisciplinary disciplines such as bioinformatics in particular. The upside of the current situation is that the Polish economy so far has proven strong enough to defend itself against the global crisis, and science funding in Poland has not experienced major cuts unlike in many other European countries.

Poland is already a member of several international programs and organizations that are relevant to bioinformatics, such as the European Programme of Cooperation in the field of Scientific and Technological Research (COST) or the European Molecular Biology Organization (EMBO). However, thus far Poland is not yet a member of ELIXIR, a major European initiative toward the development of pan-European research infrastructure for managing the data deluge that is too vast for any single institution or country to handle. It is hoped that in the preparations for the next financial perspective of the EU for 2014–2020, the scientific and organizational successes of Polish bioinformatics will be taken into account by policy- and decision makers, and that the bottom-up efforts of the computational biology community will meet with strong support from the government to develop nationwide educational and training programs and build new major research infrastructure. Although much remains to be done, the Polish bioinformatics community has demonstrated that is has reached a critical mass to tackle major challenges. We hope that in this article we have provided evidence that many modern research institutions in Poland mentioned here are attractive places to train in bioinformatics and carry out computational biology research, and for talented postdocs with appropriate experience to start their first independent groups.

**Authors' Biographies**

**Janusz M. Bujnicki** is a professor and a head of the Laboratory of Bioinformatics and Protein Engineering at the International Institute of Molecular and Cell Biology in Warsaw, and additionally leads a research group at the Bioinformatics Laboratory in the Institute of Molecular Biology and Biotechnology at the Adam Mickiewicz University in Poznań. He received his undergraduate training from Interdisciplinary Individual Studies in Mathematics and Natural Sciences at the University of Warsaw. He holds a master's degree and a PhD in biology (both from the University of Warsaw), a DSc degree in biochemistry from the Institute of Biochemistry and Biophysics, Polish Academy of Sciences (PAS), and the title of professor. He has also been elected a member of the Academy of Young Scientists at PAS. He is currently a president of the Polish Bioinformatics Society and serves as an editor of a number of scientific journals and a book series. His current research interests include the combination of theoretical and experimental research, in particular modeling and design of RNA and protein-RNA complexes, and engineering of enzymes with new specificities. He has published more than 200 scientific papers that have been cited >6,000 times according to Google Scholar, and has won numerous academic awards such as the EMBO and HHMI Young Investigator Award.

**Jerzy Tiuryn** is a full professor at the Faculty of Mathematics, Informatics and Mechanics (MIM), University of Warsaw. He is a leader of a research group in computational biology and bioinformatics at MIM. For the past two terms (2005–2012), he was a vice dean of MIM for scientific matters and international relations. His background is mathematics and computer science. He received all his scientific degrees (MS, PhD, DSc, title of professor) in computer science. Initially, his research was concentrated in theoretical computer science, including semantics and verification of programs, logics of programs, lambda calculus, and type theory. In the beginning of the twenty-first century, he changed his research interest to computational biology and bioinformatics, and started to build a research group in this area at University of Warsaw. He supervised about a dozen PhD theses, half of them in bioinformatics. He is currently interested in gene regulation, sequence analysis, and evolution. In 1996, he was elected a member of Academia Europaea (Informatics Section). He is a board member of European Research Consortium for Informatics and Mathematics (ERCIM). He was the first president and founding member of Polish Bioinformatics Society (2008–2012). He has published more than 130 scientific papers, with their total number of citations >4,800 according to Google Scholar.
